# Supplementary material for: Heparin-based hydrogel scaffolding alters the transcriptomic profile and increases the chemoresistance of MDA-MB-231 triple-negative breast cancer cells
Source: Biomater Sci. 2020 Feb 13;8(10):2786–96. doi: 10.1039/c9bm01481k (PMC7497406; doi:10.1039/c9bm01481k)
Supplement: Supplementary file 2 [file BM-008-C9BM01481K-s002.zip › Supplementary File 4/EGFvControl/Pathways/my_analysis.Gsea.1545200981068/HALLMARK_MITOTIC_SPINDLE.html]

Details for gene set HALLMARK\_MITOTIC\_SPINDLE[GSEA]

|  || Dataset | expr.class.cls#EGF\_versus\_CONTROL.class.cls#EGF\_versus\_CONTROL\_repos |
| Phenotype | class.cls#EGF\_versus\_CONTROL\_repos |
| Upregulated in class | EGF |
| GeneSet | HALLMARK\_MITOTIC\_SPINDLE |
| Enrichment Score (ES) | 0.31250143 |
| Normalized Enrichment Score (NES) | 1.4376853 |
| Nominal p-value | 0.008064516 |
| FDR q-value | 0.036576353 |
| FWER p-Value | 0.474 |
Table: GSEA Results Summary

  

Fig 1: Enrichment plot: HALLMARK\_MITOTIC\_SPINDLE      
 Profile of the Running ES Score & Positions of GeneSet Members on the Rank Ordered List

  

| PROBE | DESCRIPTION (from dataset) | GENE SYMBOL | GENE\_TITLE | RANK IN GENE LIST | RANK METRIC SCORE | RUNNING ES | CORE ENRICHMENT || 1 | AURKA | na |  |  | 163 | 2.181 | 0.0061 | Yes |
| 2 | CNTRL | na |  |  | 402 | 1.845 | 0.0060 | Yes |
| 3 | BUB1 | na |  |  | 455 | 1.797 | 0.0154 | Yes |
| 4 | TUBA4A | na |  |  | 624 | 1.689 | 0.0179 | Yes |
| 5 | TPX2 | na |  |  | 997 | 1.516 | 0.0086 | Yes |
| 6 | PRC1 | na |  |  | 1054 | 1.491 | 0.0157 | Yes |
| 7 | PLK1 | na |  |  | 1074 | 1.488 | 0.0247 | Yes |
| 8 | KIF15 | na |  |  | 1127 | 1.469 | 0.0318 | Yes |
| 9 | DOCK2 | na |  |  | 1128 | 1.469 | 0.0417 | Yes |
| 10 | KIF23 | na |  |  | 1186 | 1.445 | 0.0485 | Yes |
| 11 | CENPJ | na |  |  | 1238 | 1.430 | 0.0554 | Yes |
| 12 | DLGAP5 | na |  |  | 1276 | 1.420 | 0.0630 | Yes |
| 13 | RACGAP1 | na |  |  | 1310 | 1.409 | 0.0708 | Yes |
| 14 | BRCA2 | na |  |  | 1333 | 1.399 | 0.0790 | Yes |
| 15 | KIF11 | na |  |  | 1334 | 1.399 | 0.0885 | Yes |
| 16 | TUBGCP3 | na |  |  | 1374 | 1.387 | 0.0958 | Yes |
| 17 | SMC4 | na |  |  | 1418 | 1.372 | 0.1027 | Yes |
| 18 | LMNB1 | na |  |  | 1433 | 1.368 | 0.1112 | Yes |
| 19 | BIRC5 | na |  |  | 1448 | 1.364 | 0.1197 | Yes |
| 20 | CEP192 | na |  |  | 1479 | 1.352 | 0.1272 | Yes |
| 21 | GEMIN4 | na |  |  | 1521 | 1.340 | 0.1341 | Yes |
| 22 | SASS6 | na |  |  | 1535 | 1.337 | 0.1424 | Yes |
| 23 | NUSAP1 | na |  |  | 1541 | 1.335 | 0.1511 | Yes |
| 24 | CKAP5 | na |  |  | 1552 | 1.333 | 0.1596 | Yes |
| 25 | MAPRE1 | na |  |  | 1563 | 1.330 | 0.1680 | Yes |
| 26 | SYNPO | na |  |  | 1629 | 1.311 | 0.1734 | Yes |
| 27 | ROCK1 | na |  |  | 1702 | 1.294 | 0.1783 | Yes |
| 28 | KIF4A | na |  |  | 1715 | 1.291 | 0.1864 | Yes |
| 29 | RASAL2 | na |  |  | 1823 | 1.262 | 0.1893 | Yes |
| 30 | MYH10 | na |  |  | 1933 | 1.239 | 0.1919 | Yes |
| 31 | KIF2C | na |  |  | 1975 | 1.227 | 0.1980 | Yes |
| 32 | CENPF | na |  |  | 1986 | 1.224 | 0.2057 | Yes |
| 33 | KIF20B | na |  |  | 2009 | 1.217 | 0.2127 | Yes |
| 34 | ANLN | na |  |  | 2023 | 1.214 | 0.2202 | Yes |
| 35 | NDC80 | na |  |  | 2032 | 1.212 | 0.2280 | Yes |
| 36 | TOP2A | na |  |  | 2151 | 1.189 | 0.2298 | Yes |
| 37 | CENPE | na |  |  | 2189 | 1.181 | 0.2358 | Yes |
| 38 | EZR | na |  |  | 2225 | 1.173 | 0.2418 | Yes |
| 39 | FBXO5 | na |  |  | 2371 | 1.139 | 0.2419 | Yes |
| 40 | KIF22 | na |  |  | 2457 | 1.122 | 0.2450 | Yes |
| 41 | ESPL1 | na |  |  | 2577 | 1.097 | 0.2461 | Yes |
| 42 | SPTAN1 | na |  |  | 2725 | 1.072 | 0.2456 | Yes |
| 43 | TUBGCP5 | na |  |  | 2903 | 1.043 | 0.2433 | Yes |
| 44 | CDK1 | na |  |  | 2921 | 1.040 | 0.2494 | Yes |
| 45 | LRPPRC | na |  |  | 3083 | 1.010 | 0.2477 | Yes |
| 46 | KIF5B | na |  |  | 3136 | 1.000 | 0.2517 | Yes |
| 47 | ARHGAP10 | na |  |  | 3147 | 0.997 | 0.2579 | Yes |
| 48 | NEK2 | na |  |  | 3222 | 0.981 | 0.2606 | Yes |
| 49 | ECT2 | na |  |  | 3275 | 0.973 | 0.2645 | Yes |
| 50 | TTK | na |  |  | 3293 | 0.971 | 0.2701 | Yes |
| 51 | RANBP9 | na |  |  | 3294 | 0.971 | 0.2766 | Yes |
| 52 | KNTC1 | na |  |  | 3331 | 0.965 | 0.2813 | Yes |
| 53 | DLG1 | na |  |  | 3579 | 0.924 | 0.2745 | Yes |
| 54 | SMC1A | na |  |  | 3638 | 0.915 | 0.2776 | Yes |
| 55 | CCDC88A | na |  |  | 3746 | 0.897 | 0.2780 | Yes |
| 56 | ARHGAP29 | na |  |  | 3867 | 0.874 | 0.2776 | Yes |
| 57 | PCNT | na |  |  | 4080 | 0.839 | 0.2721 | Yes |
| 58 | CD2AP | na |  |  | 4107 | 0.836 | 0.2763 | Yes |
| 59 | CEP57 | na |  |  | 4218 | 0.819 | 0.2761 | Yes |
| 60 | FARP1 | na |  |  | 4232 | 0.817 | 0.2809 | Yes |
| 61 | ALS2 | na |  |  | 4263 | 0.813 | 0.2848 | Yes |
| 62 | CEP72 | na |  |  | 4324 | 0.804 | 0.2870 | Yes |
| 63 | RASA1 | na |  |  | 4463 | 0.783 | 0.2851 | Yes |
| 64 | KATNA1 | na |  |  | 4477 | 0.780 | 0.2896 | Yes |
| 65 | ITSN1 | na |  |  | 4520 | 0.774 | 0.2926 | Yes |
| 66 | NIN | na |  |  | 4526 | 0.774 | 0.2976 | Yes |
| 67 | SAC3D1 | na |  |  | 4537 | 0.773 | 0.3023 | Yes |
| 68 | EPB41L2 | na |  |  | 4626 | 0.759 | 0.3027 | Yes |
| 69 | VCL | na |  |  | 4660 | 0.755 | 0.3061 | Yes |
| 70 | APC | na |  |  | 4827 | 0.725 | 0.3022 | Yes |
| 71 | CNTROB | na |  |  | 5035 | 0.703 | 0.2961 | Yes |
| 72 | ALMS1 | na |  |  | 5074 | 0.697 | 0.2988 | Yes |
| 73 | CDC42 | na |  |  | 5090 | 0.695 | 0.3027 | Yes |
| 74 | CCNB2 | na |  |  | 5148 | 0.685 | 0.3043 | Yes |
| 75 | ATG4B | na |  |  | 5158 | 0.684 | 0.3084 | Yes |
| 76 | PXN | na |  |  | 5252 | 0.672 | 0.3080 | Yes |
| 77 | CLIP1 | na |  |  | 5322 | 0.661 | 0.3089 | Yes |
| 78 | SHROOM2 | na |  |  | 5400 | 0.650 | 0.3092 | Yes |
| 79 | ARHGEF12 | na |  |  | 5421 | 0.647 | 0.3125 | Yes |
| 80 | CDK5RAP2 | na |  |  | 5663 | 0.615 | 0.3040 | No |
| 81 | PPP4R2 | na |  |  | 5848 | 0.592 | 0.2983 | No |
| 82 | ARHGEF7 | na |  |  | 5849 | 0.592 | 0.3023 | No |
| 83 | SMC3 | na |  |  | 5872 | 0.588 | 0.3051 | No |
| 84 | NF1 | na |  |  | 5879 | 0.587 | 0.3087 | No |
| 85 | NOTCH2 | na |  |  | 6144 | 0.549 | 0.2985 | No |
| 86 | YWHAE | na |  |  | 6474 | 0.506 | 0.2846 | No |
| 87 | MID1 | na |  |  | 6528 | 0.499 | 0.2852 | No |
| 88 | RFC1 | na |  |  | 6554 | 0.495 | 0.2872 | No |
| 89 | CAPZB | na |  |  | 6861 | 0.461 | 0.2742 | No |
| 90 | DYNLL2 | na |  |  | 7054 | 0.439 | 0.2671 | No |
| 91 | PCM1 | na |  |  | 7062 | 0.439 | 0.2697 | No |
| 92 | CLASP1 | na |  |  | 7088 | 0.435 | 0.2713 | No |
| 93 | ARHGEF11 | na |  |  | 7097 | 0.434 | 0.2738 | No |
| 94 | PIF1 | na |  |  | 7206 | 0.421 | 0.2709 | No |
| 95 | TUBD1 | na |  |  | 7214 | 0.420 | 0.2734 | No |
| 96 | RAB3GAP1 | na |  |  | 7351 | 0.402 | 0.2689 | No |
| 97 | TBCD | na |  |  | 7424 | 0.394 | 0.2678 | No |
| 98 | PDLIM5 | na |  |  | 7457 | 0.391 | 0.2688 | No |
| 99 | KLC1 | na |  |  | 7470 | 0.390 | 0.2707 | No |
| 100 | TLK1 | na |  |  | 7475 | 0.389 | 0.2732 | No |
| 101 | SSH2 | na |  |  | 7559 | 0.378 | 0.2713 | No |
| 102 | RASA2 | na |  |  | 7751 | 0.356 | 0.2637 | No |
| 103 | FGD4 | na |  |  | 7758 | 0.355 | 0.2658 | No |
| 104 | PCGF5 | na |  |  | 7773 | 0.354 | 0.2674 | No |
| 105 | RALBP1 | na |  |  | 7868 | 0.341 | 0.2648 | No |
| 106 | KIF3B | na |  |  | 7929 | 0.334 | 0.2639 | No |
| 107 | CDC27 | na |  |  | 7997 | 0.325 | 0.2625 | No |
| 108 | ARHGDIA | na |  |  | 8010 | 0.324 | 0.2641 | No |
| 109 | KIF1B | na |  |  | 8020 | 0.323 | 0.2658 | No |
| 110 | PAFAH1B1 | na |  |  | 8291 | 0.294 | 0.2535 | No |
| 111 | ABI1 | na |  |  | 8474 | 0.275 | 0.2458 | No |
| 112 | RAPGEF6 | na |  |  | 8673 | 0.250 | 0.2371 | No |
| 113 | ARFGEF1 | na |  |  | 8721 | 0.244 | 0.2363 | No |
| 114 | CTTN | na |  |  | 8987 | 0.214 | 0.2238 | No |
| 115 | OPHN1 | na |  |  | 9014 | 0.213 | 0.2238 | No |
| 116 | NCK1 | na |  |  | 9082 | 0.204 | 0.2217 | No |
| 117 | PKD2 | na |  |  | 9218 | 0.191 | 0.2159 | No |
| 118 | KATNB1 | na |  |  | 9366 | 0.177 | 0.2093 | No |
| 119 | INCENP | na |  |  | 9447 | 0.164 | 0.2062 | No |
| 120 | PALLD | na |  |  | 9524 | 0.154 | 0.2033 | No |
| 121 | RHOT2 | na |  |  | 9543 | 0.152 | 0.2033 | No |
| 122 | KPTN | na |  |  | 9637 | 0.145 | 0.1994 | No |
| 123 | WASF1 | na |  |  | 9807 | 0.125 | 0.1914 | No |
| 124 | CDC42BPA | na |  |  | 9923 | 0.113 | 0.1861 | No |
| 125 | HOOK3 | na |  |  | 10517 | 0.052 | 0.1552 | No |
| 126 | LATS1 | na |  |  | 10540 | 0.048 | 0.1544 | No |
| 127 | RABGAP1 | na |  |  | 10668 | 0.029 | 0.1479 | No |
| 128 | UXT | na |  |  | 10693 | 0.026 | 0.1468 | No |
| 129 | ARF6 | na |  |  | 10802 | 0.014 | 0.1412 | No |
| 130 | STAU1 | na |  |  | 11181 | -0.025 | 0.1215 | No |
| 131 | ABL1 | na |  |  | 11459 | -0.057 | 0.1073 | No |
| 132 | CEP250 | na |  |  | 11677 | -0.082 | 0.0965 | No |
| 133 | ARAP3 | na |  |  | 12347 | -0.162 | 0.0624 | No |
| 134 | STK38L | na |  |  | 12408 | -0.171 | 0.0604 | No |
| 135 | NCK2 | na |  |  | 12425 | -0.174 | 0.0607 | No |
| 136 | BCAR1 | na |  |  | 12438 | -0.176 | 0.0613 | No |
| 137 | TSC1 | na |  |  | 12942 | -0.238 | 0.0364 | No |
| 138 | CDC42EP1 | na |  |  | 13118 | -0.260 | 0.0290 | No |
| 139 | KIFAP3 | na |  |  | 13177 | -0.270 | 0.0277 | No |
| 140 | BIN1 | na |  |  | 13406 | -0.303 | 0.0178 | No |
| 141 | RICTOR | na |  |  | 13580 | -0.327 | 0.0109 | No |
| 142 | MYO9B | na |  |  | 13828 | -0.355 | 0.0003 | No |
| 143 | TAOK2 | na |  |  | 13961 | -0.371 | -0.0042 | No |
| 144 | SEPT9 | na |  |  | 13997 | -0.378 | -0.0035 | No |
| 145 | RAPGEF5 | na |  |  | 14038 | -0.383 | -0.0030 | No |
| 146 | ARHGAP4 | na |  |  | 14158 | -0.399 | -0.0066 | No |
| 147 | EPB41 | na |  |  | 14182 | -0.403 | -0.0051 | No |
| 148 | SHROOM1 | na |  |  | 14712 | -0.482 | -0.0296 | No |
| 149 | MAP3K11 | na |  |  | 14735 | -0.485 | -0.0275 | No |
| 150 | WASL | na |  |  | 14807 | -0.498 | -0.0279 | No |
| 151 | WASF2 | na |  |  | 15125 | -0.534 | -0.0410 | No |
| 152 | LLGL1 | na |  |  | 15178 | -0.542 | -0.0401 | No |
| 153 | ARFIP2 | na |  |  | 15315 | -0.568 | -0.0434 | No |
| 154 | SOS1 | na |  |  | 15328 | -0.569 | -0.0402 | No |
| 155 | MARK4 | na |  |  | 15359 | -0.576 | -0.0379 | No |
| 156 | MAP1S | na |  |  | 15411 | -0.584 | -0.0367 | No |
| 157 | ARL8A | na |  |  | 15625 | -0.611 | -0.0438 | No |
| 158 | CSNK1D | na |  |  | 15899 | -0.665 | -0.0536 | No |
| 159 | TUBGCP6 | na |  |  | 15920 | -0.670 | -0.0502 | No |
| 160 | FGD6 | na |  |  | 15943 | -0.676 | -0.0468 | No |
| 161 | AKAP13 | na |  |  | 16185 | -0.728 | -0.0546 | No |
| 162 | FSCN1 | na |  |  | 16186 | -0.728 | -0.0497 | No |
| 163 | CDC42EP4 | na |  |  | 16212 | -0.734 | -0.0460 | No |
| 164 | CDC42EP2 | na |  |  | 16218 | -0.736 | -0.0413 | No |
| 165 | CYTH2 | na |  |  | 16349 | -0.769 | -0.0430 | No |
| 166 | DOCK4 | na |  |  | 16444 | -0.795 | -0.0426 | No |
| 167 | BCL2L11 | na |  |  | 16498 | -0.810 | -0.0399 | No |
| 168 | BCR | na |  |  | 16554 | -0.825 | -0.0373 | No |
| 169 | MYO1E | na |  |  | 16746 | -0.868 | -0.0415 | No |
| 170 | ARHGEF3 | na |  |  | 16801 | -0.886 | -0.0383 | No |
| 171 | ABR | na |  |  | 16940 | -0.933 | -0.0393 | No |
| 172 | ARHGEF2 | na |  |  | 16955 | -0.937 | -0.0337 | No |
| 173 | ARHGAP27 | na |  |  | 17014 | -0.955 | -0.0304 | No |
| 174 | SUN2 | na |  |  | 17023 | -0.958 | -0.0243 | No |
| 175 | PLEKHG2 | na |  |  | 17246 | -1.020 | -0.0291 | No |
| 176 | TUBGCP2 | na |  |  | 17325 | -1.051 | -0.0262 | No |
| 177 | NUMA1 | na |  |  | 17337 | -1.054 | -0.0196 | No |
| 178 | HDAC6 | na |  |  | 17451 | -1.104 | -0.0181 | No |
| 179 | MID1IP1 | na |  |  | 17594 | -1.147 | -0.0179 | No |
| 180 | MARCKS | na |  |  | 17808 | -1.228 | -0.0208 | No |
| 181 | KIF3C | na |  |  | 17992 | -1.324 | -0.0215 | No |
| 182 | RHOF | na |  |  | 18083 | -1.373 | -0.0170 | No |
| 183 | GSN | na |  |  | 18275 | -1.477 | -0.0171 | No |
| 184 | ARHGAP5 | na |  |  | 18337 | -1.520 | -0.0101 | No |
| 185 | CLIP2 | na |  |  | 18338 | -1.520 | 0.0001 | No |
| 186 | NEDD9 | na |  |  | 18380 | -1.552 | 0.0084 | No |
| 187 | NET1 | na |  |  | 18490 | -1.636 | 0.0137 | No |
| 188 | TIAM1 | na |  |  | 18560 | -1.701 | 0.0215 | No |
| 189 | SORBS2 | na |  |  | 18626 | -1.778 | 0.0301 | No |
Table: GSEA details [plain text format]

  

Fig 2: HALLMARK\_MITOTIC\_SPINDLE      
 Blue-Pink O' Gram in the Space of the Analyzed GeneSet

  

Fig 3: HALLMARK\_MITOTIC\_SPINDLE: Random ES distribution      
 Gene set null distribution of ES for **HALLMARK\_MITOTIC\_SPINDLE**
